# DNA damage as a consequence of NLR activation

**DOI:** 10.1371/journal.pgen.1007235

**Published:** 2018-02-20

**Authors:** Eleazar Rodriguez, Jonathan Chevalier, Hassan El Ghoul, Kristoffer Voldum-Clausen, John Mundy, Morten Petersen

**Affiliations:** Department of Biology, University of Copenhagen. Copenhagen, Denmark; The Sainsbury Laboratory, UNITED KINGDOM

## Abstract

DNA damage observed during plant immune responses is reported to be an intrinsic component of plant immunity. However, other immune responses may suppress DNA damage to maintain host genome integrity. Here, we show that immunity-related DNA damage can be abrogated by preventing cell death triggered by Nucleotide-binding, Leucine-rich-repeat immune Receptors (NLRs). SNI1 (suppressor of *npr1-1*, inducible 1), a subunit of the structural maintenance of chromosome (SMC) 5/6 complex, was reported to be a negative regulator of systemic acquired resistance (SAR) and to be necessary for controlling DNA damage. We find that cell death and DNA damage in *sni1* loss-of-function mutants are prevented by mutations in the NLR signaling component *EDS1*. Similar to *sni1*, elevated DNA damage is seen in other autoimmune mutants with cell death lesions, including *camta3*, *pub13* and *vad1*, but not in *dnd1*, an autoimmune mutant with no visible cell death. We find that as in *sni1*, DNA damage in *camta3* is EDS1-dependent, but that it is also NLR-dependent. Using the NLR RPM1 as a model, we also show that extensive DNA damage is observed when an NLR is directly triggered by effectors. We also find that the expression of DNA damage repair (DDR) genes in mutants with cell death lesions is down regulated, suggesting that degraded DNA that accumulates during cell death is a result of cellular dismantling and is not sensed as damaged DNA that calls for repair. Our observations also indicate that SNI1 is not directly involved in SAR or DNA damage accumulation.

## Author summary

DNA is constantly subjected to damaging agents that can cause mutations, disease and cell death. Not surprisingly, cells have evolved mechanisms to repair damaged DNA and preserve its integrity. Recent reports suggested that the innate immune system in plants might actively damage host DNA as a mechanism to boost resistance to pathogens. This process was shown to be mediated by the phytohormone Salicylic Acid and the DNA damage repair protein and immunity regulator SNI1. In contrast, we report that the DNA damage observed in *sni1* is independent of its role in DNA damage repair and is instead linked to autoimmunity and shared with other, unrelated autoimmune mutants. We demonstrate, genetically, that cellular dismantling triggered by NLR-mediated immunity is a more plausible explanation for the DNA damage observed in those mutants. Our results provide clarification and new insight into the role of DNA damage and immunity.

## Introduction

Plants rely on a dual layered innate immune system to defend against pathogens. In a first layer, pattern recognition receptors (PRRs) detect pathogen associated molecular patterns (PAMPs), leading to PAMP triggered immunity (PTI) [1]. To avoid PTI and establish infection, pathogens deliver effectors to modify host proteins. In a second immune layer, cytoplasmic Nucleotide-binding, Leucine-rich-repeat Receptors (NLRs) may detect these modifications and activate immunity. Some plant NLRs thus ‘guard’ host ‘guardees’ and activate effector triggered immunity (ETI) [2,3]. NLR activation and ETI are dependent on signaling components including ENHANCED DISEASE SUSCEPTIBILITY 1 (EDS1) and NON RACE-SPECIFIC DISEASE RESISTANCE (NDR1) [4].

ETI can be accompanied by a type of programmed cell death at infection sites called the hypersensitive response (HR) [5]. HR cell death shares features with apoptosis, including changes in membrane integrity, cysteine protease mediated cleavage of key proteins, and DNA degradation [6–8]. Following local cell death, systemic acquired resistance (SAR) can be induced to boost immunity in uninfected parts of the plant. HR and SAR are regulated by the phytohormone salicylic acid (SA), whose accumulation promotes defense responses. Consequently, interference with SA accumulation impairs defense responses [9].

Numerous *Arabidopsis* autoimmune mutants exhibit accumulation of SA and inappropriate activation of immunity in the absence of infection [2,10]. These mutants can be broadly divided into those with de-repressed, SA-dependent defense responses and those that also exhibit accelerated cell death [2]. Importantly, autoimmunity in many such mutants can be suppressed by mutations in *EDS1*, *NDR1*, or specific *NLRs* [11–13]. In line with this, Lolle et al. [14] recently reported a screen to systematically abrogate autoimmunity in selected mutants by disruption of NLR function [14].

DNA damage repair (DDR) is essential to maintain genome stability, and compelling evidence links DNA damage responses with innate immune programs in mammals [15] and plants [16]. Foreign and damaged host DNA, including DNA breaks generated by viral integration, can trigger innate immune responses [16–18]. Thus, both alien and damaged host DNA function as danger signals that can alert the immune system. Interestingly, DNA damage accompanying infection was reported to be an intrinsic component of plant immunity. For example, SA pretreatment that reportedly caused DNA damage was concluded to boost immune responses [19]. This conclusion was supported by the finding that loss-of-function mutants of SNI1, a subunit of the DDR complex SMC5/6 earlier indicated as negative regulator of SAR [20], accumulated significant levels of DNA damage under normal growth conditions [19]. This increased DNA damage was proposed to be caused by decreased DDR activity in the absence of SNI1. In contrast, Song and Bent [21] demonstrated that DNA damage was induced by pathogen infection and that the plant immune system tried to diminish this damage to preserve genome integrity. While these and other studies indicate that DNA damage may trigger immune responses, it seems unclear whether DNA damage is actively induced in infected host cells or is a consequence of infection.

Here we report that activation of NLR signalling and ETI are sufficient to trigger DNA damage accumulation observed during plant immune responses. We demonstrate this using autoimmune mutants that display accumulation of DNA damage in the absence of pathogen infection. We show that such DNA damage is abrogated by shutting down NLR mediated signaling, and thus immunity. We also provide evidence that DNA damage accumulation observed in *sni1* mutants is not due to faulty DDR but is dependent on NLR signaling components.

## Results

### DNA damage accumulates in autoimmune mutants with macroscopic cell death lesions

**Fig 1:**
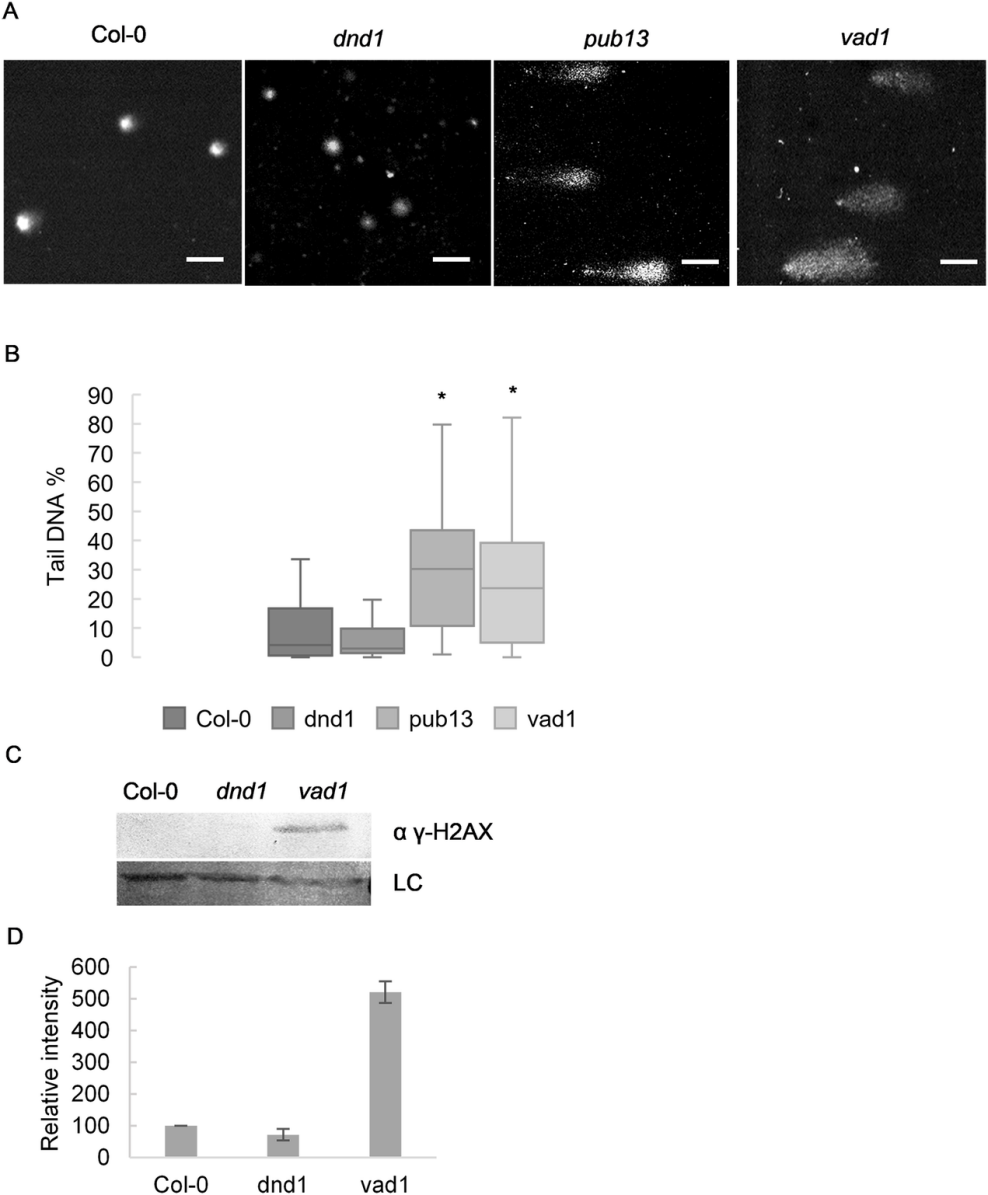
Mutants with runaway cell death accumulate DNA damage in uninfected conditions. *pub13* and *vad1* mutants have more DNA damage than Col-0 or *dnd1*. (A) Representative images of comets and (B) % tail DNA quantification of the genotypes. Values of 3 biological replicates made of pools of different individuals (at least 50 comets scored per biological replicate). Bars marked with different letters are statistically different (*P*≤ 0.01) among samples according to a Holm-Sidak multiple comparison test. (C) Immunoblot of histone extraction from Col-0, *dnd1* and *vad1* probed with anti γ-H2AX antibody. Unspecific band was used as loading control. (D) Quantification of the immunoblot of (C) γ-H2AX analysis normalized to input and to Col-0 (set to 100,Values are mean ± SD of 2 biological replicates).

To investigate if accumulation of DNA damage is a common feature among mutants with constitutive immune activation, we performed the alkaline version of the single cell gel electrophoresis (Comet assay) to estimate the amount of DNA damage in autoimmune mutants including *pub13*, *vad1* and *dnd1*. The alkaline version of the comet assay is capable of detecting DNA double-strand breaks, single-strand breaks, alkali-labile sites, DNA-DNA/DNA-protein cross-linking, and incomplete excision repair sites [22]. *PUB13* (*Plant U-Box 13*) encodes an E3 ubiquitin ligase implicated in ubiquitination and degradation of the PRR FLS2 [23], *VAD1* (*Vascular Associated Death 1*) encodes a membrane-bound protein [24], and *DND1* (*Defense No Death 1*) encodes a cyclic nucleotide gated channel [25] While *pub13*, *vad1* and *dnd1* all over accumulate SA, only *pub13* and *vad1* also exhibit accelerated cell death. We found that *vad1* and *pub13* had more DNA damage (*P*<0.05) than wild type (Fig 1A and 1B). Interestingly, the level of DNA damage observed in *dnd1* was not significantly different from the level in wild type (Fig 1B). However, it should be mentioned that *dnd1* was reported to display macroscopic cell death when grown under certain conditions, and it is thus possible that in other conditions it would also display elevated DNA damage. We also performed an immunoblot against the phosphorylated version of Histone 2AX (γ-H2AX), a common marker for DNA double strand breaks, which corroborated our comet assay data, *i*.*e*. while *vad1* strongly accumulated γ-H2AX, this was not detected in Col-0 or *dnd1* (Fig 1C and 1D). These results point to a connection between macroscopic cell death and DNA damage, and provide indirect evidence that increased SA levels may not be the major reason for DNA damage accumulation in autoimmune mutants.

### Accumulation of DNA damage is dependent on the NLR signaling component EDS1

**Fig 2:**
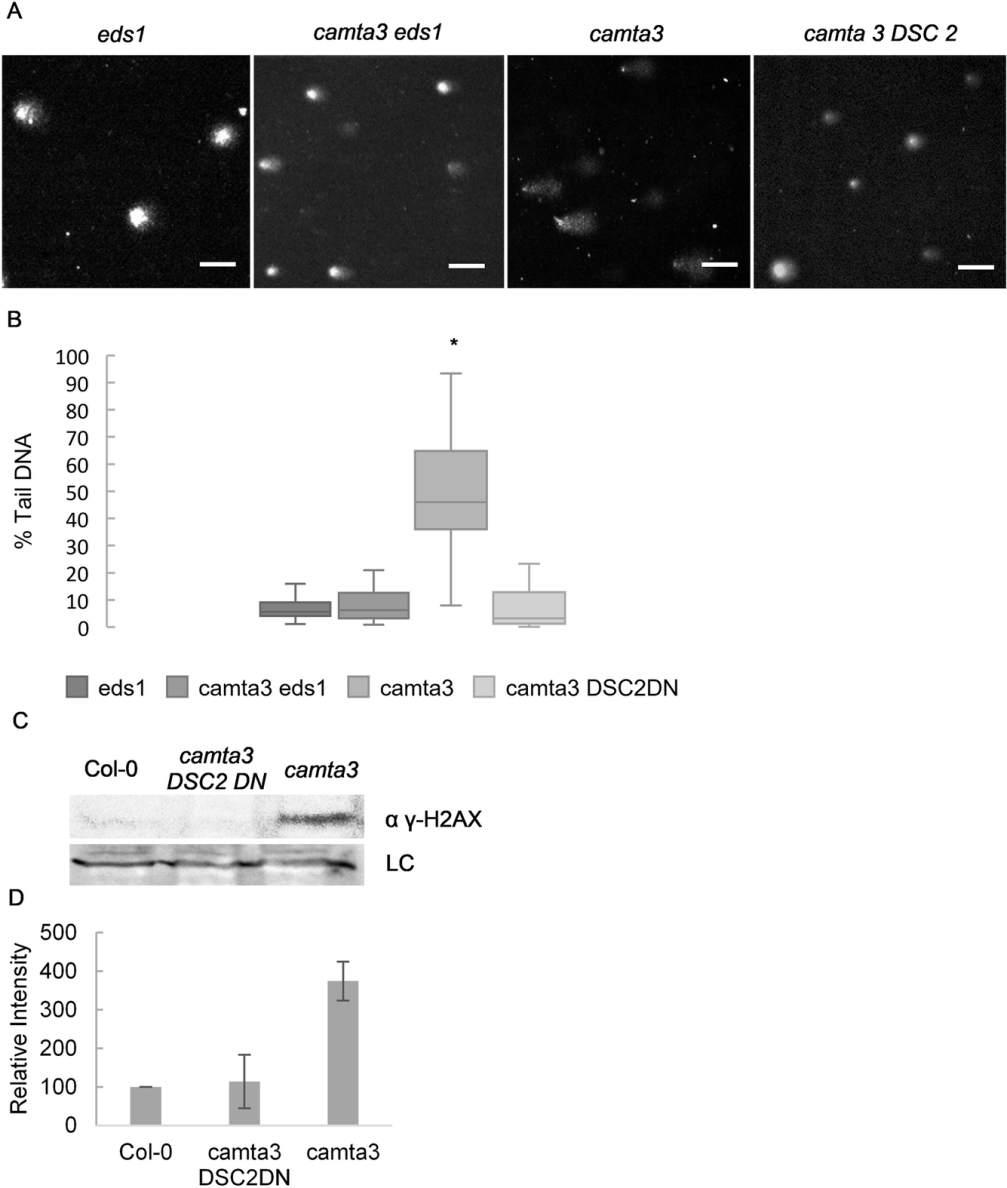
DNA damage accumulation in the *camta 3* mutant is dependent on NLR signalling. Accumulation of DNA damage in *camta 3–1* is dependent on the NLR signalling component EDS1 and on the NLR DSC2. (A) Representative pictures of comets and (B) % tail DNA quantification of the genotypes. Values are of 3 biological replicates made of pools of different individuals (at least 50 comets scored per biological replicate). Bars marked with different letters are statistically different (*P*≤ 0.01) among samples according to a Holm-Sidak multiple comparison test. (C) Immunoblot of histone extraction from Col-0, *camta3* and *camta3* expressing DN-DSC2 probed with anti γ-H2AX antibody. Unspecific band was used as loading control. (D) Quantification of the immunoblot of (C) γ-H2AX analysis normalized to input and to Col-0 (set to 100, values are mean ± SD of 2 biological replicates).

Many autoimmune mutant phenotypes can be partly or fully rescued by loss-of-function of key immune signaling proteins such as EDS1 or NDR1 [2]. We speculated that DNA damage accumulation in autoimmune mutants might also be dependent on such signaling components. To address this, we compared the levels of DNA damage in another autoimmune mutant, *camta3*, caused by loss-of-function of the CAMTA3 calmodulin-binding transcription factor [26] to *camta3 eds1-2* double mutants. This showed that introducing *eds1-2* into the *camta3-1* background completely rescues the DNA damage accumulation observed in the *camta3-1* single mutant (Fig 2A and 2B). We recently reported that transgenic expression of dominant negative (DN) forms of *Arabidopsis* NLRs specifically disrupt the function of the corresponding wild type alleles [14]. That study showed that a *DN* mutant of an *NLR* named *Dominant suppressor of camta3 2* (*DSC2S*) fully suppressed autoimmunity in *camta3* [14]. Consequently, we also did the comet assay with *camta3-1* expressing *DN-DSC2* and observed that DNA damage accumulation was reduced to control levels (Fig 2A and 2B). Immunoblotting of γ-H2AX showed that *camta 3* accumulation of this DSB marker is mediated by the NLR DSC2 (Fig 2C and 2D). These results indicate that DNA damage accumulation in *camta3* is dependent on an intact NLR signaling pathway and the induction of immunity triggered by DSC2.

**Fig 3:**
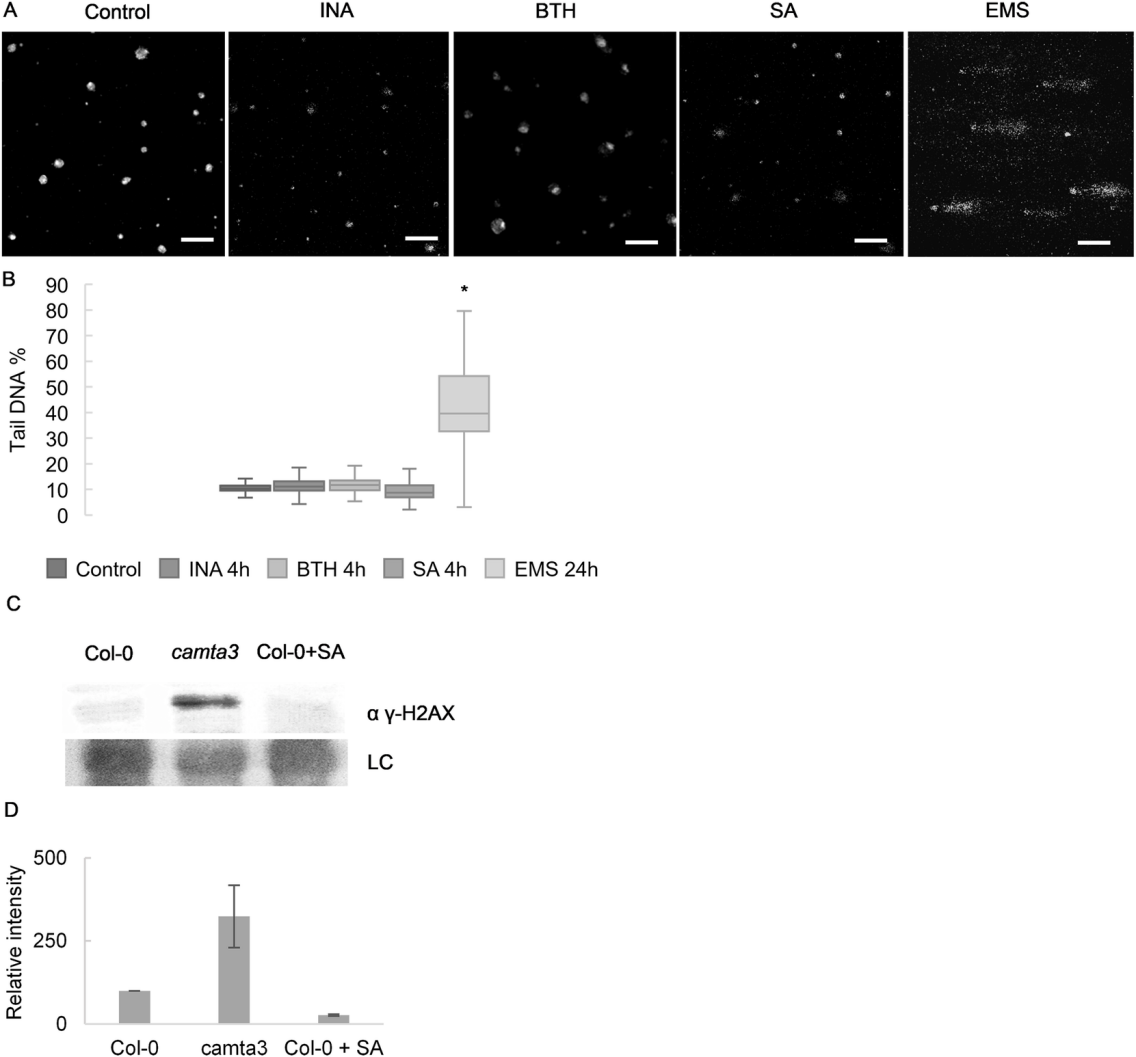
SA analogues BTH and INA do not induce significant accumulation of DNA damage. Col-0 plants treated with SA, INA or BTH do not display significant DNA damage accumulation when compared to untreated plants. (A) Representative pictures of comets and (B) % tail DNA quantification of the conditions described. Values of 3 biological replicates made of pools of different individuals (at least 50 comets scored per biological replicate). Bars marked with different letters are statistically different (*P*≤ 0.01) among samples according to a Holm-Sidak multiple comparison test. (C) Immunoblot of histone extraction from Col-0, *camta3* and Col-0 + 1mM SA probed with anti γ-H2AX antibody. Ponceau staining was used as loading control. (D) Quantification of the immunoblot of (C) γ-H2AX analysis normalized to input and to Col-0 (set to 100) (Values are mean ± SD of 2 biological replicates).

DNA damage accumulation thus seems to be a common feature of autoimmune mutants with accelerated cell death including pub*13*, *vad1* and *camta3*. Our data also suggest that constitutive accumulation of SA is insufficient to cause DNA damage since *dnd1* mutants have no signs of increased DNA damage. This conclusion is based on the observation that all the mutants tested accumulate SA but only *camta3*, *vad1* and *pub13* have macroscopic cell death lesions [24–28] and DNA damage. In contrast to a previous report [17], Song and Bent [21], could not detect significantly increased DNA damage in WT plants treated with SA, and we verified this with SA and its analogs BTH and INA (Fig 3A–3D).

### ETI activation leads to accumulation of damaged DNA even in the absence of pathogens

**Fig 4:**
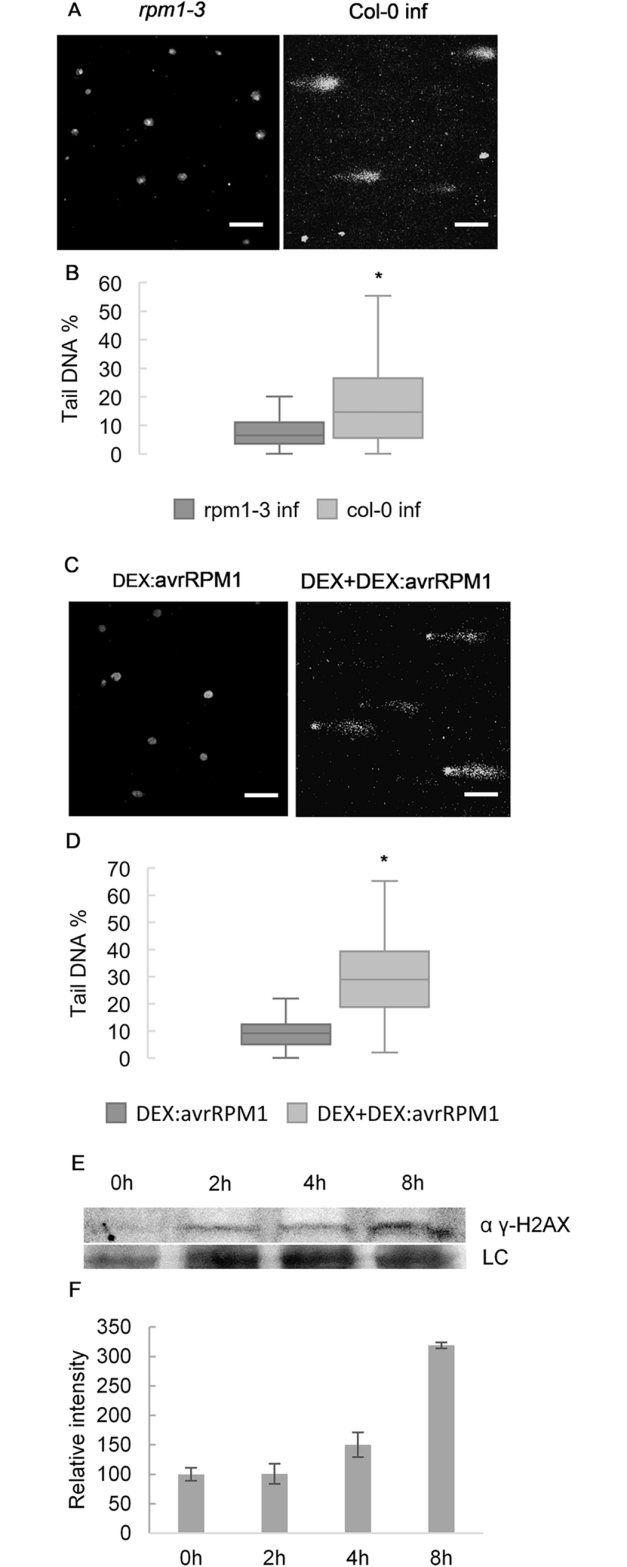
ETI activation leads to DNA damage accumulation even in the absence of pathogens. Recognition of a single effector (avrRPM1) is sufficient to induce DNA damage accumulation. (A and B) Col-0 accumulated more DNA damage than *rpm1-3* mutants infected with *P*. *fluorescens* harboring avrRPM1. (C and D) *In planta* expression of avrRPM1 under control of a DEX inducible promotor is sufficient to cause DNA damage (8h after treatment). (A and C) Representative pictures of comet assays and (B and D) Tail DNA % quantification of the genotypes and conditions described. Values of 3 biological replicates made of pools of different individuals (at least 50 comets scored per biological replicate). Bars marked with different letters are statistically different (*P*≤ 0.01) among samples according to a Holm-Sidak multiple comparison test. (E) Immunoblot of samples of plants sprayed with DEX after the given time points probed with anti γ-H2AX antibody. (F) Quantification of the immunoblot of (C) γ-H2AX analysis normalized to input and to 0h sample (set to 100) (Values are mean ± SD of 2 biological replicates).

NLRs are thought to guard host proteins against tampering by microbial effectors, and many NLRs require EDS1 for signaling. Because the *camta3-1* phenotype is dependent on EDS1 and DSC2, we tested if detection of a single effector would be sufficient to induce accumulation of DNA damage. Song and Bent [21] showed that *P*. *fluorescens*, a bacterium known to induce systemic resistance in plants, does not cause DNA damage accumulation when infiltrated into *Arabidopsis*. We therefore infected *rpm1-3*, a loss-of-function mutant of the RPM1 NLR which detects AvrRPM1, and wild type Col-0 with *P*. *fluorescens* expressing the effector *AvrRPM1*. As expected, while Col-0 triggers ETI and accumulates DNA damage upon recognition of avrRPM1, the *rpm1-3* mutant does not (Fig 4A and 4B). We corroborated this by estimating DNA damage in plants expressing *AvrRPM1* under the control of a DEX inducible promoter. While DEX treatment did not induce DNA damage accumulation in wild type Col-0, plants expressing DEX-induced *AvrRPM1* had higher levels of DNA damage compared to their untreated counterparts (Fig 4C and 4D). This experiment demonstrates that DNA damage can be induced by triggering an NLR pathway based on the recognition of a single effector. Thus, in this case, DNA damage is first found after the induction of immunity. We then wanted to determine if DNA damage observed was part of an early response to effector recognition. To this end we performed a time course in DEX-induced *AvrRPM1* expressing plants and verified that γ-H2AX accumulated upon DEX induction and was more than doubled after 8h (Fig 4E and 4F).

### Immunity related phenotypes of *sni1* are dependent on EDS1

**Fig 5:**
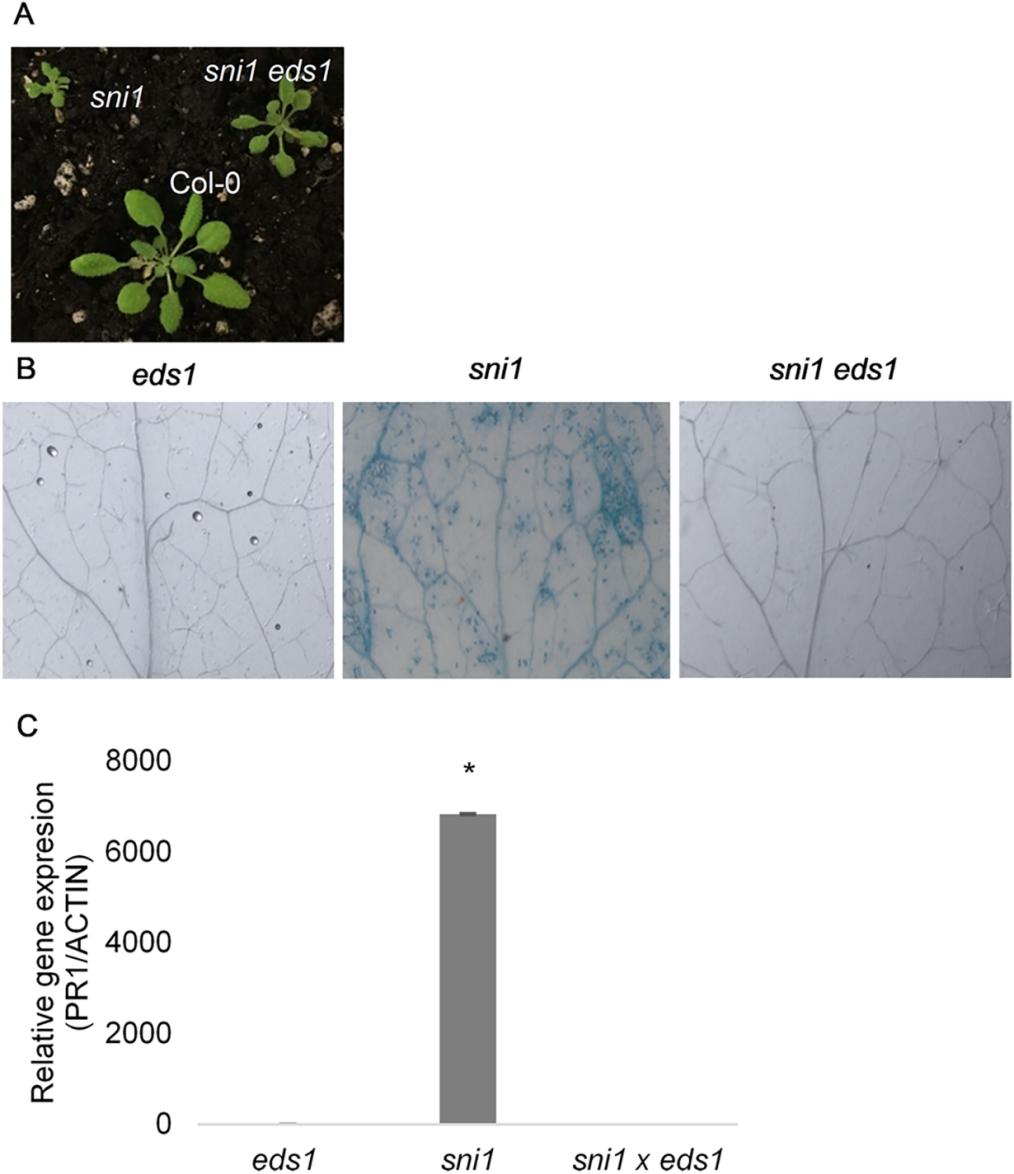
*sni1* autoimmune phenotype is dependent of EDS1. (A) picture of 5 week-old plants grown under short day conditions displaying partial rescue of *sni1* dwarfism in *sni1 eds1* (8h days). (B) Trypan blue staining of 2 week-old *sni1*, *sni1 eds1-2* and *eds 1–2* plants showed that run-away cell death in *sni1* is dependent on EDS1. (C) PR1 relative transcript accumulation in *sni1* was abrogated in the *sni1 eds1-2* double mutant. Results, normalized to UBQ10 and relative to Col-0, are shown as mean ± SD of 3 biological replicates.

**Fig 6:**
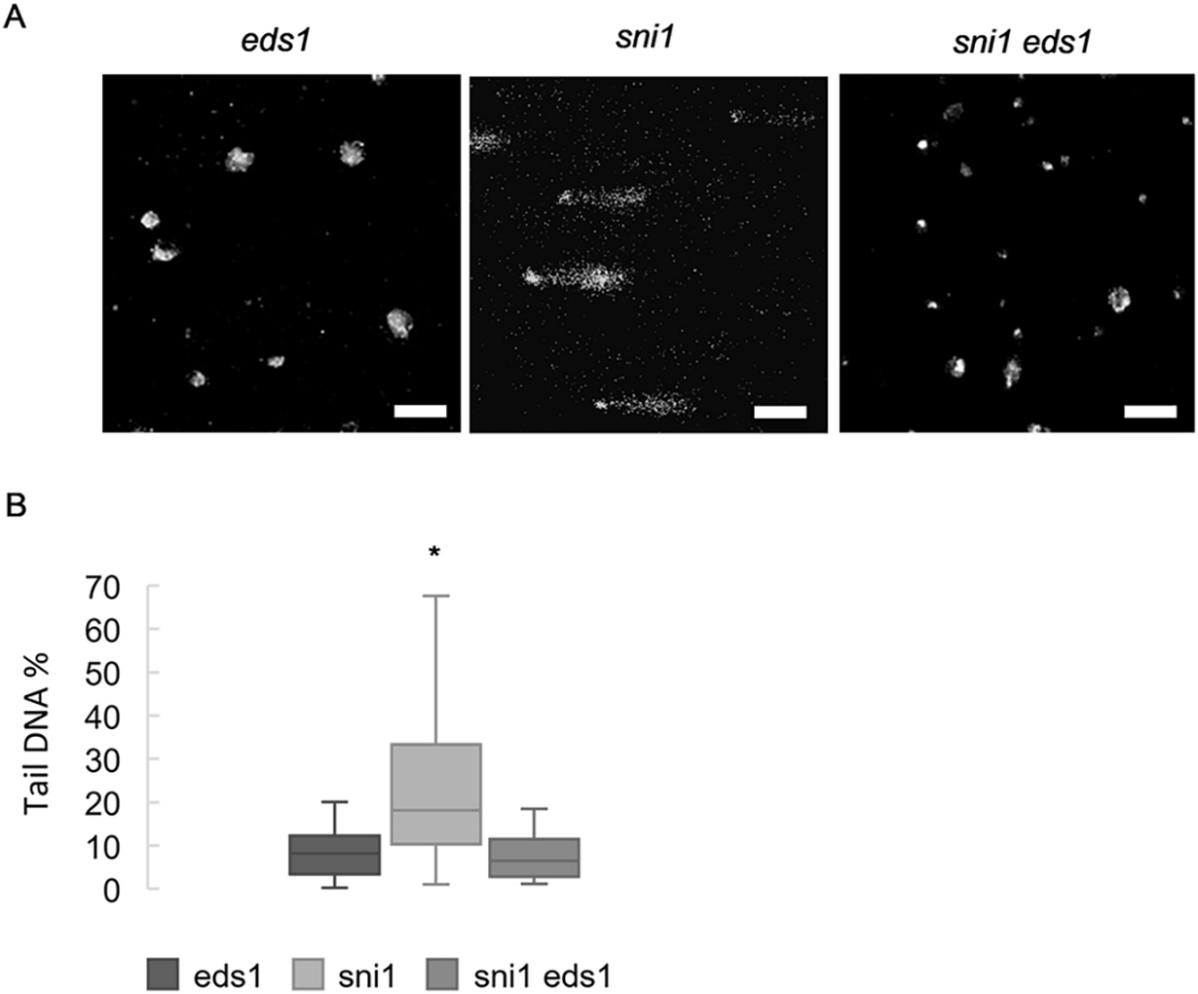
DNA damage accumulation in *sni1* is caused by autoimmunity. (A and B) DNA damage accumulation in *sni1*, *sni1 eds1-2* and *eds1-2*. (A) Representative pictures of comets and (B) % tail DNA quantification of the genotypes. Values of 3 biological replicates made of pools of different individuals (at least 50 comets scored per biological replicate). Bars marked with different letters are statistically different (*P*≤ 0.01) among samples according to a Holm-Sidak multiple comparison test.

Since *sni1* is an autoimmune mutant that exhibits accelerated cell death [19,20], we tested if *sni1* could be rescued by shutting down immunity. To this end, we crossed *sni1* to *eds1-2* and verified that the doubly homozygous plants had their growth partially restored when compared to *sni1* (Fig 5A). Furthermore, cell death (by trypan blue staining) and *PR1* transcript accumulation of transcripts of marker *PATHOGENESIS-RELATED 1* (*PR1*) were completely abrogated in *sni1 eds1-2* plants (Fig 5B and 5C). These results, together with comet assay data from *sni1* and *sni1 eds1-2* (Fig 6A and 6B), confirmed that DNA damage accumulation in *sni1* is due to autoimmunity and not to defective DNA damage repair [19].

### DDR machinery is shut down upon activation of ETI

**Fig 7:**
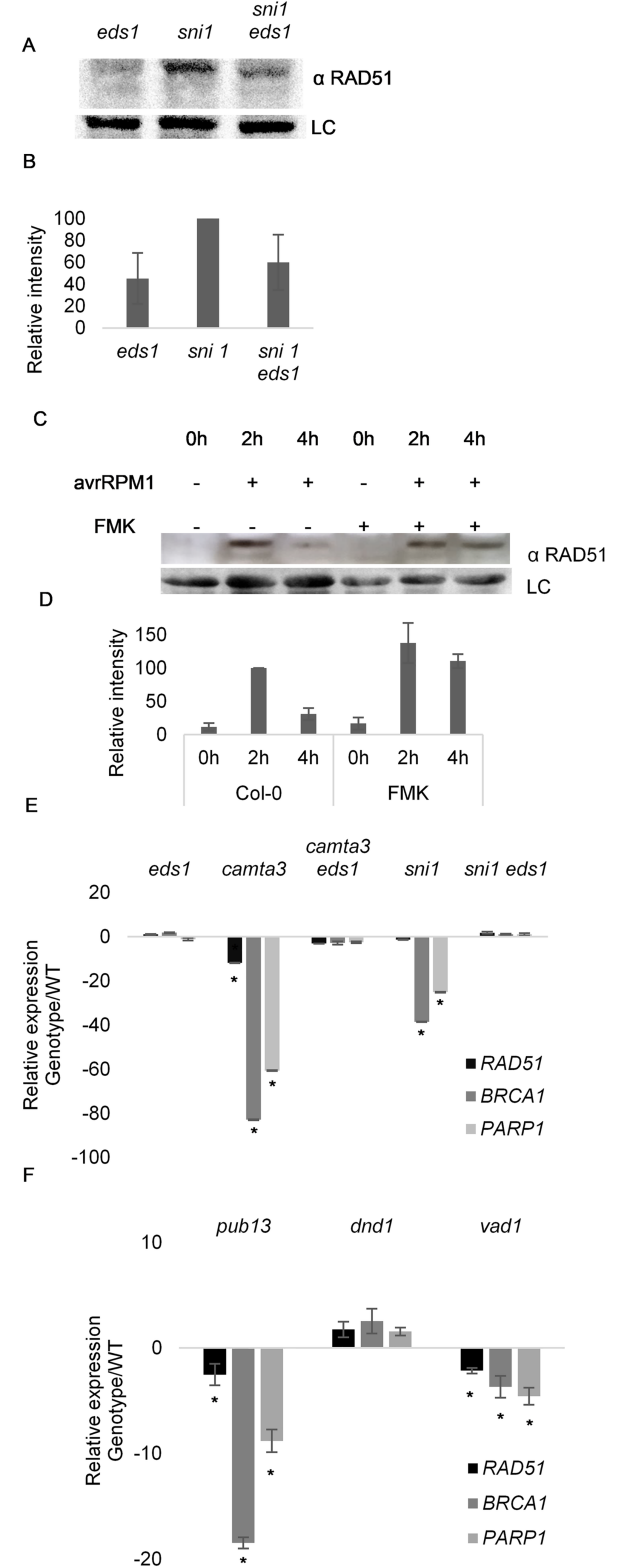
DNA Damage Repair shutdown is dependent on ETI signalling components. (A to D) RAD51 accumulates in an immunity dependent manner. (A) *sni1* displays enhanced accumulation of RAD51 which is reduced in *sni1 eds1-2*. Immunoblot of total protein extracts from *eds1-2*, *sni1*, *sni1 eds1-2* probed with anti RAD51 antibody. An unspecific band was used as loading control. (B) Quantification of the immunoblot of (B) RAD51 normalized to input and to Col-0 (set to 100) (Values are mean ± SD of 3 biological replicates). (C) ETI activation causes RAD51 degradation by proteases with Caspase 3-like activity. Protein extracts from plants subjected to the conditions given were probed with anti RAD51 antibody. Unspecific band was used as loading control. (D) Quantification of the immunoblot of (C) RAD51 normalized to input and to Col-0 infected at 2h (set to 100) (Values are mean ± SD of 3 biological replicates). (E and F) DDR genes are downregulated during HR PCD, (E) downregulation of DDR genes in *camta 3–1* and *sni1* is dependent of EDS1. (F) *vad1* and *pub 13*, but not *dnd1*, display DDR gene downregulation. Gene expression was normalized to UBQ10 and is relative to Col-0 (values represent the average ± SD of 3 biological replicates).

SNI1 was proposed to be a negative regulator of RAD51, a key DDR gene involved in double strand break repair, because RAD51 accumulates in *sni1* mutants [29]. Since *sni1* phenotypes are suppressed by mutation of *EDS1*, we also tested if the involvement of SNI in RAD51 regulation could be linked to *sni1* autoimmunity. Using the same antibody as Wang et al. [29], we observed that accumulation of RAD51 in *sni1* mutants was diminished in the *sni1 eds1* double mutant (Fig 7A and 7B). This result again points to an immunity related origin for *sni1* phenotypes.

In mammals, activation of apoptosis leads to Caspase 3 mediated cleavage of RAD51 to inactivate the DNA damage repair machinery [30,31]. We therefore tested if *At*RAD51 was cleaved during effector triggered immunity, and if such cleavage could be affected by Caspase 3 inhibitors. To this end, we infiltrated Col-0 plants with *P*. *syringae* AvrRPM1 in the presence or absence of the Caspase 3 inhibitor Z-DEVD-FMK, which was recently shown to inhibit protease activity in *Arabidopsis* [7]. Infection with *P*. *syringae* led to rapid accumulation of RAD51 (Fig 7C and 7D) 2 hours post infection (hpi) for all conditions tested. With the establishment of ETI (4 hpi) only co-infiltration with Z-DEVD-FMK stabilized RAD51. This observation that RAD51 is degraded upon induction of ETI is in keeping with the shutdown of DDR responses during apoptosis [30,31] and the accumulation of γ-H2AX seen in Fig 4E.

Since it is reasonable to assume that cells shut down DDR when undergoing programmed cell death such as that during the HR in plants, we also analyzed the relative transcript accumulation of a subset of DDR genes in *sni1* and other autoimmune cell death mutants. While DDR genes were previously shown to be upregulated in *sni1* [19], we found that several DDR genes were downregulated in *sni1* (Fig 7E). Such genes were also downregulated in other autoimmune mutants with accelerated cell death (Fig 7E and 7F), but not in *dnd1* which does not exhibit cell death (Fig 7F). In addition, the apparent reduction in the levels of DDR gene transcripts in *sni1* and *camta3* were dependent on EDS1 (Fig 7E). These results again indicate that the suppression of DDR in *sni1* is caused by NLR signaling.

## Discussion

A model has been proposed in which pathogen infection induces SA accumulation which leads to increased DNA damage that acts as an intrinsic component of plant immune responses [19]. This model is based on observations that SA treatment induced DNA damage, and that DNA damage accumulated in uninfected loss-of-function mutants of SNI1 encoding a subunit of the SMC5/6 complex required for controlling DNA damage. In contrast, we (Fig 3) find that SA or its analogues BTH and INA do not cause an increase in DNA damage. Similarly, Song and Bent [21] found that SA treatment prior to pathogen infection reduced the accumulation of damaged DNA. We note that application of 1mM SA can be phytotoxic [32] and could consequentially cause DNA damage accumulation under certain growth conditions. We also demonstrate here that other immune-related cell death mutants accumulate DNA damage. Such damage is therefore not an exclusive feature of the *sni1* mutant. Notably, the *dnd1* mutant, which over-accumulates SA but exhibits ‘Defense No Death’, does not accumulate damaged DNA. This indicates that processes involved in immune-related cell death, rather than constitutive defense responses, cause DNA damage. Immune-related cell death encompasses DNase mediated oligonucleosomal DNA fragmentation which is normally seen as DNA laddering [33]. The comet assay, which is able to detect DNA strand breaks, would thus also ‘score’ oligonucleosomal DNA fragmentation as damaged DNA. This may explain the accumulation of putatively damaged DNA in autoimmune mutants.

Our analyses of infections with *P*. *syringae* avrRPM1, and of plants expressing DEX inducible avrRPM1, further confirm that NLR triggered cell death is sufficient to induce DNA damage accumulation, even in the absence of a pathogen (Fig 3A and 3B). Since avrRPM1 is not recognized in the *rpm1-3* mutant, *rpm1-3* fails to trigger ETI and consequently does not accumulate significant amounts of damaged DNA as measured by the Comet assay. Thus, it is the host immune system that in this case causes DNA damage. We note that we do not rule out the possibility that pathogens, or their activities, may cause DNA damage, as it is well described in other systems that diverse pathogens affect host genome integrity [34].

Importantly, mutations in the NLR signaling component EDS1 completely suppress DNA damage accumulation, as measured by the comet assay, in both the *sni1* (Fig 6) and *camta3* (Fig 2) autoimmune mutants. Likewise, expression of a dominant negative mutant form of the NLR DSC2 is sufficient to prevent DNA damage accumulation in the single *camta3-1* mutant. Thus, the DNA damage seen in these autoimmune mutants is indirect. That such damage occurs in the four unrelated autoimmune mutants described here supports a model in which DNA damage is a consequence of cell death.

It could be argued than in an alternative model for *sni1*, defective DNA damage repair causes DNA damage accumulation which in turn induces upregulation of immune responses, *e*.*g*. activation of NLRs due to damaged DNA. In this model, however, the double *sni1 eds1* mutant should retain the DNA damage accumulation seen in the single *sni1* mutant while losing all of the enhanced immune responses. Because DNA damage accumulation is restored to basal levels in the double mutant we maintain that DNA damage in these mutants is a consequence of autoimmunity.

SNI1 was originally identified in a screen for suppressors of NPR1, a known positive regulator of SAR. Because *sni1* mutants restore *PR1* gene expression and pathogen resistance in *npr1* backgrounds, SNI1 was proposed to be a negative regulator of SAR. However, neither macroscopic nor microscopic cell death was originally reported in *sni1*, even after INA treatment [20]. Surprisingly, *sni1* was later reported to exhibit cell death in the absence of pathogens [19]. We also find that *sni1* displays cell death (Fig 4B) and, more importantly, that increased *PR1* expression, stunted growth, and HR PCD in *sni1* are dependent on the NLR signaling component EDS1 (Fig 4A–4C). Autoimmunity in *sni1* may therefore be better explained by a guard model in which SNI1 and/or other components related to the SMC5/6 complex are guarded by an NLR(s).

While it is still possible that SNI1 plays a role in immune responses, these effects are overshadowed by *EDS1*-dependency. For example, partial suppression of *sni1* growth defects by *eds1* could be due to an intermediate phenotype between *eds1* mutants (which can be larger than wild type plants) and *sni1*, and therefore not directly linked to autoimmunity.

A potential caveat to a *SNI1* guard model is that mutations in the upstream DDR components *RAD17* and *ATR* rescue the *sni1* phenotype [19]. An explanation could be that the NLR(s) which may recognize *sni1* loss-of-function needs to be associated with other components of the SMC complex to become activated and trigger immune responses. If so, such components or the complex may be so severely altered or absent in *sni1 rad17* or *sni1 atr* double mutants as to abrogate the function of the NLR guard. Tangential support of a model in which the whole SMC5/6 complex is guarded comes from the finding that the mutant of MMS21, another member of the SMC5/6 complex, also displays stunted growth, spontaneous cell death and accumulation of damaged DNA [35]. Future work could characterize double *mms21 eds1* and *mms21 atm/atr double* mutants to check if the *mms21* phenotype is suppressed, as with *sni1*. It is also possible that, like RAD51 and BRCA2, SNI1 could be positively involved in immunity by maintaining genome integrity during infection. This would make *sni1* and other components of the DDR machinery potential targets for pathogen effectors and thus likely candidates for guarding by NLRs.

Considering the involvement of SNI1 in RAD51 regulation, our observation that transcripts of DDR genes are downregulated in *sni1* (Fig 7E and 7F) again fits with a model in which autoimmunity, and not a regulatory function on SNI1, affects the levels of DDR transcripts and RAD51 protein. In contrast, Yan et al. and Xu et al. [19,35] observed increased DDR gene transcripts in *mms21* and *sni1*. An explanation for these differences is that they used 2 week-old plants, while we used plants at a more advanced developmental stage (6 week-old) to allow the onset of runaway cell death in some of the mutants tested. At early developmental stages, a constitutive defense phenotype would lead to an increase in DDR which would later be switched off as plant tissues start to succumb to HR PCD. In addition, Wang et al. [29] showed that RAD51 and BRCA2 are actively recruited and bind to the promotors of defense related genes during SAR. This could explain the initial upregulation of these genes in young *sni1* and *mms21* plants. The recruitment of the DDR machinery to defense genes during SAR may be necessary to protect actively transcribed regions of the genome, or it may be a strategy to prevent pathogens from tampering with defense responses by interfering with genome integrity. It is well established in humans that pathogens can affect host genome integrity [34], so it is probable that DDR genes which maintain genome integrity would be upregulated during initial stages of defense. However, once the balance between life and death has shifted towards the latter, the DDR machinery is shutdown to allow for cellular dismantling.

In conclusion, we demonstrate that activation of NLR-mediated immunity leads to DNA damage accumulation as an effect of the execution of HR PCD. We provide evidence of *sni1* autoimmunity and propose that this autoimmunity underlies some previous misconceptions about the function of SNI1 as a negative regulator of SAR, its involvement in RAD51 regulation, and the accumulation of DNA damage in *sni1* loss-of-function mutants.

## Materials and methods

### Plant growth conditions

Sterilized seeds were placed on soil supplemented with vermiculite, perlite, and fertilizer. Plants were grown in chambers at 21°C under 8 hours of light and 16 hours of darkness. The mutants *camta 3–1* (SALK_00152), *vad1* (SALK_00782), *pub13* (SALK_093164) and *sni1* (SAIL_298_H07) were obtained from the European *Arabidopsis* Stock Center (NASC) and genotyped (primers listed in Table 1). *camta 3–1 x* DSC2-DN (*At5g18370)* mutants were obtained as described in [14].

**Table 1:** Primers list for qPCR and genotyping.

**qPCR primer**	**Sequence**
RAD51-F	ATG AAG AAA CCC AGC AC
RAD51-R	TGA ACC CCA GAG GAA C
PARP1-F	TTG ACG CCA GTA GGA A
PARP1-R	AAT ACC AGC CCA GTT AG
BRCA1-F	TTG CTC AGG GCT CAC A
BRCA1-R	GGT CCT TTT GCA GGC T
Ubiquitin-F	CAC ACT CCA CTT GGT CTT GCG T
Ubiquitin-R	TGG TCT TTC CGG TGA GAG TCT TCA
PR1 F	GTAGGTGCTCTTGTTCTTCCC
PR1 R	CACATAATTCCCACGAGGATC
**Genotyping Primers**	
SNI1-F	TTC ATA CAC TTG ATT TCG GGG
SNI1-R	TCG TTT TCT TCT TTG GTG CTG
LB3 (SAIL)	CTG AAT TTC ATA ACC AAT CTC GAT ACA C
EDS1-F	TTC TTG CCC AAT TGG ATC CCA G
EDS1-R	CGG ATC CCG AAT TCT TTA GAG

### Comet sssay

Comet assays were performed as described by [22]. In brief, tissue was finely cut with a new scalpel in 300 μl of Tris Buffer (0,4 M pH 7,5) in the dark on ice. The nuclear suspension obtained was mixed 1:1 with 1% low melting point (LMP) agarose, added to a pre-coated slide (1% agarose) and incubated at 4°C for 10 mins. Afterwards, nuclear unwinding was done in alkaline solution (200 mM NaOH, 1 mM EDTA pH >13) for 20 minutes, and the slides were then electrophoresed in alkaline solution (300 mM NaOH, 1 mM EDTA, pH >13) at 0.7 V/cm for 20 mins. The slides were then neutralized, washed with Tris Buffer followed by water, then stained with SYBR Green I (Invitrogen, California, USA). Comet images were captured with a Zeiss Axioplan microscope with a CoolSnap camera CF (Photometrics, Arizona US) or with a Zeiss LSM 700 upright confocal microscope. DNA damage quantification was performed with Open Comet plug-in for ImageJ.

### Bacterial infection, SA analog treatments and protease inhibitor assay

Col-0 and *rpm1-3* leaves were syringe infiltrated with *Pst* DC3000 (*AvrRpm1*) strain (1 × 10^8^ CFU mL^−1^) or with *P*. *fluorescens* (1x10^7^ CFU mL^−1^) in 10mM MgCl_2_. For SA analog assays, Col-0 plants were sprayed with 100 μM BTH (Syngenta, UN 3077), 100 μM INA (Sigma), 1mM SA (Sigma) or water and analyzed 4 or 24h after exposure. For the protease inhibitor assay, 10 μM Z-DEVD-FMK (Santa Cruz was infiltrated with or without *Pst* DC3000 (*AvrRpm1*).

### Trypan blue staining

Leaves were excised from 2 week-old plants from the genotypes given and stained with Lactophenol-Trypan blue, followed by distaining in chloral hydrate as described previously [36].

### Immunoblotting

Total proteins were extracted as described in [29]. In brief, tissue was flash frozen and protein extracted in 50 mM Tris-HCl (pH 7,5), 150 mM NaCl, 5 mM EDTA 0,1% Triton x-100; 0,2% Nonidet P-40, 1 mM PMSF, 1 cOmplete ULTRA Tablet, Mini, EDTA-free, (Roche) and 3 x SDS buffer was added. The extract was centrifuged at 16.000 ×g for 10 min at 4°C.The supernatant was transferred to a new tube and centrifuged twice more before SDS/PAGE analysis on a 12% gels. The samples were transferred onto nitrocellulose membrane, blocked with 5% BSA in TBS-T and sequentially probed with rabbit polyclonal Anti-Rad51 (Abcam ab63801) and anti-rabbit horseradish-conjugated antibody (Promega, W4028).

Histone extraction was performed as previously described [37]. In brief, 3 grams of tissue were ground in nuclear isolation buffer (15 mM PIPES, pH 6.8; 5 mM MgCl_2_; 0.25 M sucrose; 15 mM NaCl, 1 mM CaCl_2_; 0.8% triton X-100; 1 mM PMSF; 0.7 μg/ml pepstatin A; 30 mM NaF; 60 mM KCl, 1 tablet of cOmplete ULTRA Tablets, Mini, EDTA-free, and 1 tablet of PhosSTOP from Sigma). The liquid was passed through Miracloth and then spun at 10.000 x g for 20 mins at 4°C. The pellet was resuspended and incubated in 0.4 M H_2_SO_4_ at 4°C for at least 1h. Samples were then spun at 15.000 x g for 5 mins at 4°C. Histones were precipitated with acetone at -20°C overnight. Samples were then spun at 16.000 x g for 5 mins at 4°C. After centrifugation, the pellet was resuspended in 4M urea. Protein samples were subjected to SDS-PAGE, blotted and immunodetected with rabbit anti-human γ-H2AX antibody at 1∶1000 dilution (Sigma-Aldrich, St. Louis, MO).

Band intensity quantification was done with ImageJ, normalizing specific bands to input control.

### RNA extraction & quantitative real-time PCR (qPCR)

Total RNA was extracted using TRI reagent (Sigma) and performed according to the manufacturer’s recommendations. 1μg of total RNA was used for DNase treatment with TURBO DNA-free Kit (Ambion Life technologies). cDNA synthesis was then performed using RevertAid First Strand cDNA Synthesis Kit (Thermo Fisher) according to the manufacturer’s recommendations. qPCR was done using the Luminaris SYBR ROX qPCR Master Mix (ThermoFisher) and expression level was normalized to UBQ10 (primers listed in Table 1).
